# Cellulite: Current Understanding and Treatment

**DOI:** 10.1093/asjof/ojad050

**Published:** 2023-06-21

**Authors:** Allen Gabriel, Vivian Chan, Marissa Caldarella, Tanya Wayne, Erin O’Rorke

## Abstract

Cellulite is an aesthetically distressing skin condition that manifests as dimples and depressions, producing an uneven surface to the skin. Occurring in 80% to 90% of females, mostly on the thighs, buttocks, and hips, it is associated with profound negative psychosocial and quality of life issues. Its ethiopathogenesis and pathophysiology are likely to be multifactorial and complex and not fully understood. There is no effective treatment for cellulite, although a number of different treatment modalities are available, from noninvasive to minimally invasive. The efficacy of most treatments is unpredictable and improvements in cellulite appearance are short lived, although significant progress has been made with newer treatments. This review provides an update on the current state of knowledge about cellulite, with an emphasis on patient assessment and an individualized treatment approach for optimal results.

**Level of Evidence: 5:**

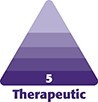

Cellulite is a dermatologic condition that predominantly affects postpubertal females.^1–3^ It is characterized by topographic changes of the skin, especially in areas of greater fat storage, mainly the thighs, buttocks, and hips. Clinically, the topographic changes manifest as dimpling, denting, or nodulation, leading to an uneven surface of the skin. The dimpling gives the skin the appearance of “mattress-like,” “cottage cheese,” or “orange peel,” which is the characteristic clinical appearance of cellulite. Medically, cellulite is referred by various terms, including gynoid lipodystrophy, nodular liposclerosis, edematofibrosclerotic panniculopathy, adiposis edematosa, dermopanniculosis deformans, and status protrusus cutis,^1^ reflecting some of the perceived pathophysiology of this condition.

Although a painless condition, because it is aesthetically unappealing, cellulite is associated with profound negative psychosocial effects. Body dissatisfaction, psychosocial distress, anxiety, and decreased quality of life are highly prevalent among females with cellulite.^4,5^ Consequently, many females seek treatment for cellulite. In 2021, over 86,000 minimally invasive cellulite treatments were performed by surgeons from the American Society of Aesthetic Plastic Surgeons.^6^

There are numerous treatments for cellulite, from noninvasive to minimally invasive modalities.^3^ However, the treatment of cellulite remains a challenge, partly because it is a complex disorder with an enigmatic etiopathogenesis and partly because of the limited efficacy of available treatments. This review provides an update on the current state of knowledge about cellulite, with an emphasis on patient assessment and an individualized treatment approach for optimal results.

## EPIDEMIOLOGY

Very little is known about the prevalence and incidence of cellulite as well as the predisposing factors for cellulite development because of a paucity of robust epidemiologic data. Despite this shortcoming, it is widely reported in the literature that an estimated 80% to 90% of postpubertal females are affected by this condition.^2,3,7–9^ Females of all races/ethnicities are affected, although Caucasian females are more susceptible than Asian or African American females.^2,3^ There is no particular age of onset for cellulite. It can occur at any age postpuberty, although it mostly appears between the ages of 20 and 30.^2,3^

Males are rarely affected by cellulite. In about 2% of males, cellulite may develop due to androgen deficiency secondary to castration, hypogonadism, Klinefelter's syndrome, or estrogen or antiandrogen therapy for prostate cancer.^2,3,9^

## ETIOPATHOGENESIS

**Figure 1.**
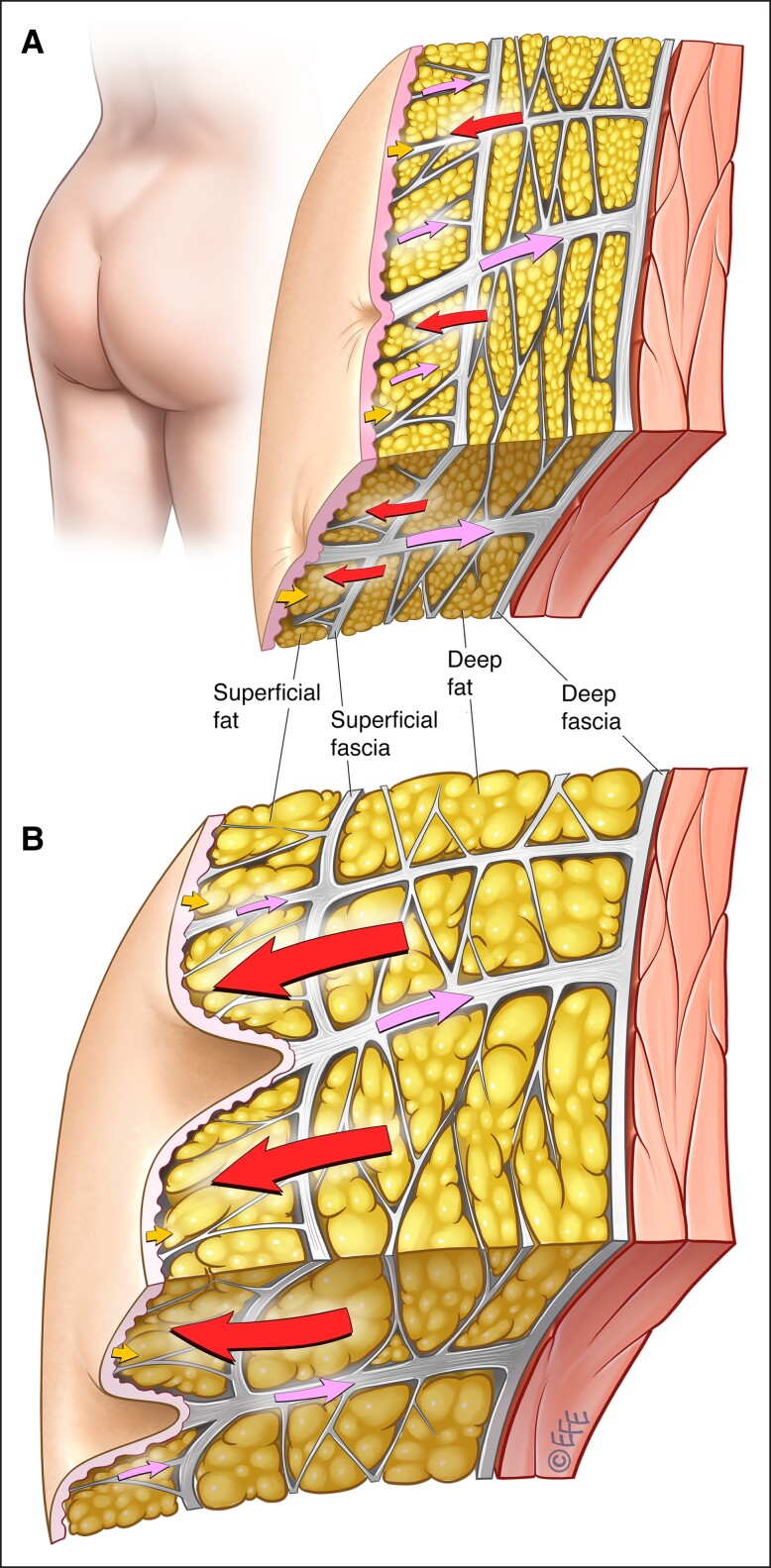
Subcutaneous gluteal architecture in females.^10^ Structure and arrangement of skin and subcutaneous tissue in individuals with (A) low-to-normal BMI or (B) high BMI. The arrows demonstrate the interplay of biomechanical forces (red arrows: outward force of fat lobules; lavender arrows: inward tethering force of the septal network, with illustrated dimorphism between the numerous short and thin septa [small lavender arrows] vs the fewer long and thick septa, which have greater stability [large lavender arrows]; yellow arrows: inward containment force of the dermis). BMI, body mass index. Artwork created by and published with permission from Dr Levent Efe, CMI.

The etiopathogenesis and pathophysiology of cellulite have not yet been fully elucidated. But an anatomical study of cadavers has provided some insights.^10^ Based on this study, from an anatomic standpoint, cellulite may be regarded as an architectural disorder of the dermis and the associated subcutaneous tissue (Figure 1).

In the gluteal region where cellulite most commonly occurs, the subcutaneous tissue consists of 5 different tissue layers (the dermis, superficial fat, superficial fascia, deep fat, and deep fascia) and 2 types of fibrous collagenase septa (short, thin septa and the tall, thick septa). The short, thin septa connect the superficial fascia to the dermis and the long, thick septa connect the deep fascia to the dermis. The short septa are more numerous than the long septa but are less stable while the long septa are less numerous but have greater stability.

The fat lobules in both adipose layers are arranged in a honeycomb-like structure and are enclosed by fibrous connective tissue with the fibrous septa interdigitating the lobules. The superficial fat globules are significantly higher in number but are smaller in height and width compared with the fat lobules in the deep fatty layer. Thus, the deep fatty layer is significantly thicker than the superficial fatty layer. An increase in body mass index (BMI) is associated with an increase in the thickness of both fatty layers: an increase in the height of the superficial layer and an increase in the number of fat lobules in the deep layer.

According to the architectural disorder hypothesis, imbalances in the inherent biomechanical forces within the septa, adipose layers, and the dermis contribute to cellulite development. The septa (short and long) impart an inward tethering force, while the adipose tissue layers impart an outward force, and the dermis imparts an inward containment force. As the short septal connections are less stable, they are unable to contain the superficial fat lobules, causing an imbalance of forces and resulting in a dimple being created at the inflexible thick septal connection to the dermis (subdermal junction). In females with low-normal BMI, the dimple created may or may not be recognized as clinical cellulite. In females with high BMI, the height of superficial fat lobules and the thickness of both fatty layers increase, exaggerating the imbalance of forces at the subdermal junction, which alters and weakens the subdermal junction, resulting in more pronounced dimples and depressions. Fat may herniate through the long septa. Fat herniation, however, is a secondary event and not a primary cause of cellulite.

**Figure 2.**
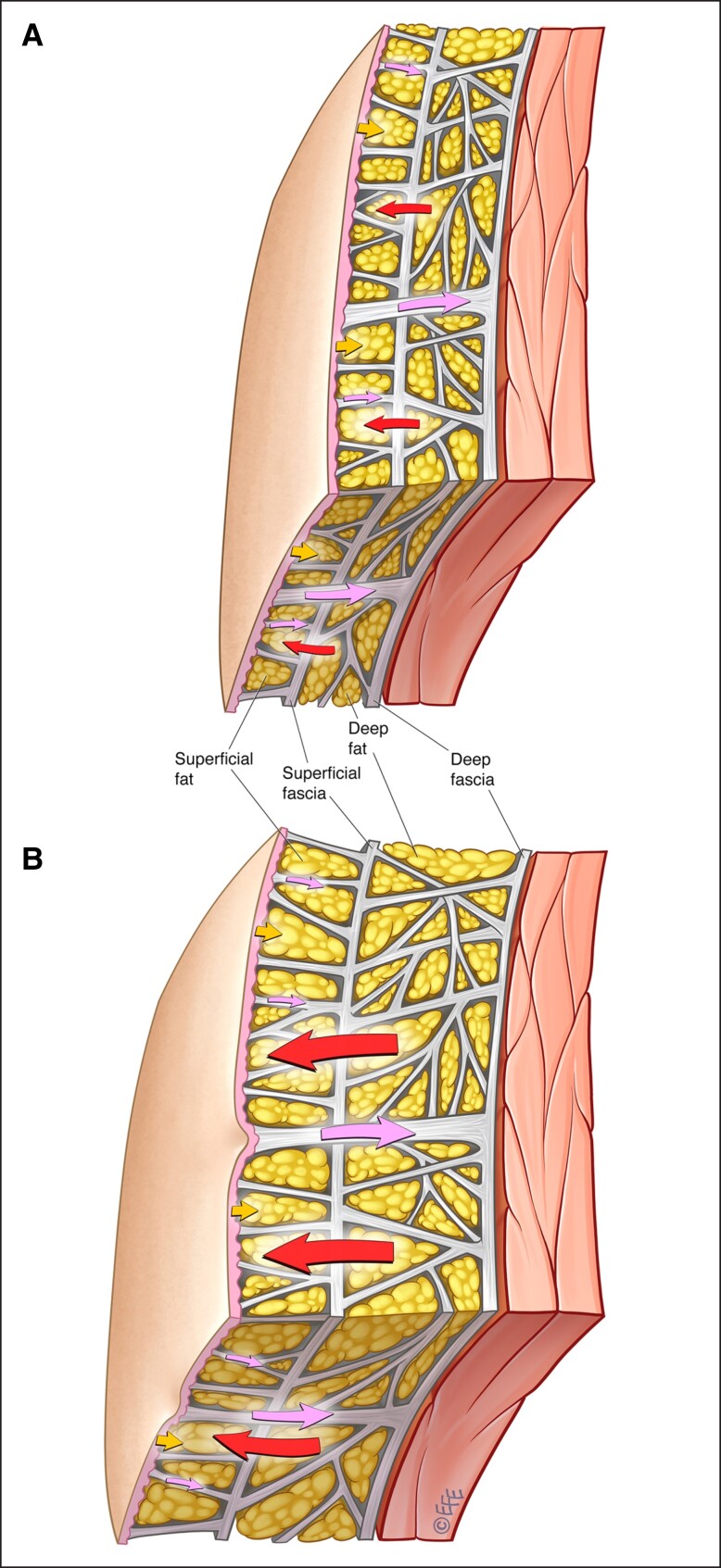
Subcutaneous gluteal architecture in males.^10^ Structure and arrangement of skin and subcutaneous tissue in individuals with low-to-normal BMI (upper panel) or high BMI (lower panel). The arrows demonstrate the interplay of biomechanical forces (red arrows: outward force of fat lobules; lavender arrows: inward tethering force of the septal network, with illustrated dimorphism between the numerous short and thin septa vs the fewer long and thick septa, which have greater stability [lavender arrows]; yellow arrows: inward containment force of the dermis). BMI, body mass index. Artwork created by and published with permission from Dr Levent Efe, CMI.

**Figure 3.**
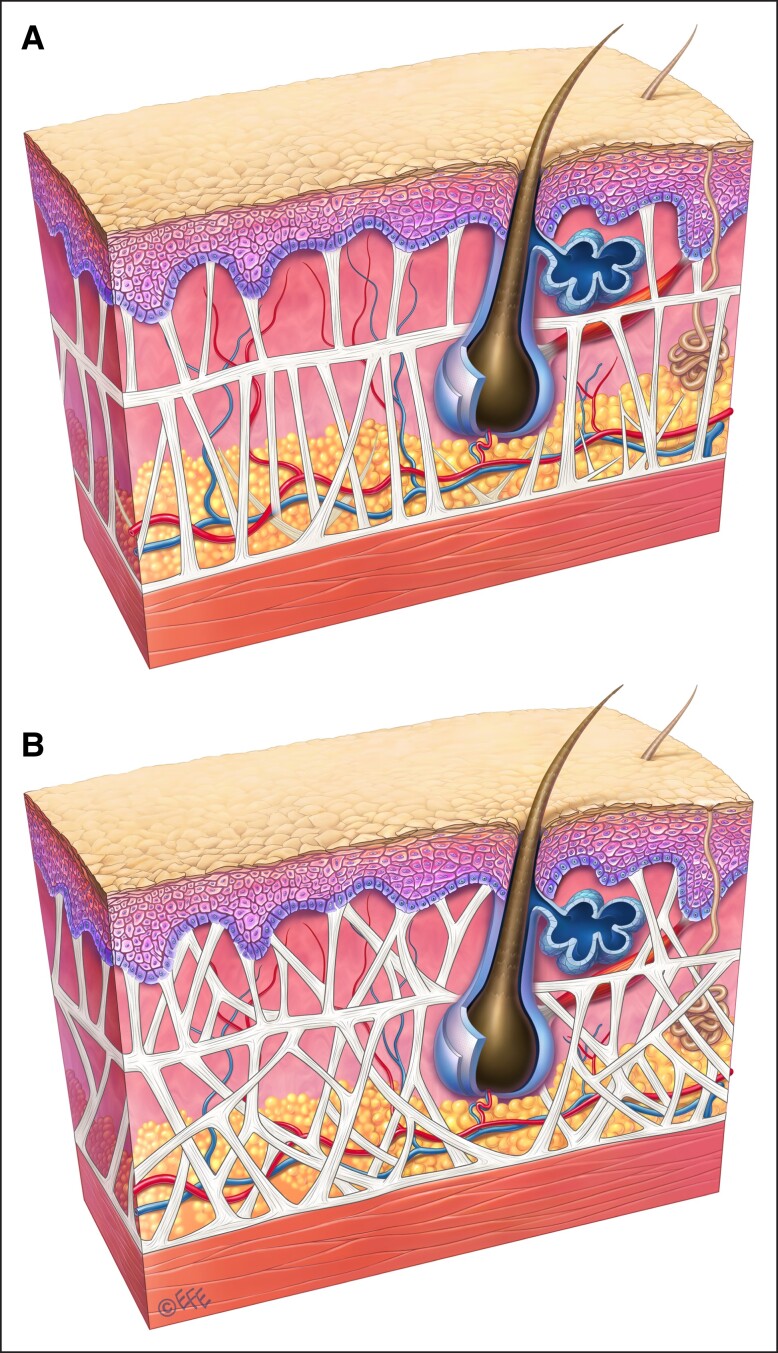
Septal dimorphism.^3^ (A) Vertical septa in females and (B) crisscrossed septa oriented at 45° angle in males. Artwork created by and published with permission from Dr Levent Efe, CMI.

Males rarely have cellulite, and this may be explained by the gender dimorphism in the fibrous septal network and the adipose layers (Figure 2).^10^ While the fibrous septa are oriented vertically to the dermis in females, they are oriented at approximately 45° angle to the dermis and crisscross in males (Figure 3).^3,11^ Males also have more numerous short, thin septa (Figure 2).^10^ Further, the septa in males are stronger and more stable than those in females. Females have fewer fat lobules and the fat lobules are greater in height and width. These sexual differences in the number and morphology of fat globules are more prominent in the superficial fatty layer than in the deep fatty layer. Thus, in a given area, males have a greater number of subcutaneous fat lobules and a greater number of septa. The greater stability and inward force provided by the higher number of septal connections in males means that males, even obese males, are less susceptible to the appearance of cellulite.

Because gender is a primary influencer of the biomechanical forces at the subdermal junction, the female sex hormone estrogen likely plays a pivotal role in the development of cellulite.^8^ In addition, high-estrogen states, such as pregnancy, nursing, chronic oral contraceptive use, or hormone replacement therapy in postmenopausal females, appear to exacerbate or worsen the progression of cellulite.

Besides the architectural disorder and gender dimorphism hypotheses, there are 2 other hypotheses for the development of cellulite: the vascular and the inflammation hypotheses. The vascular hypothesis posits that dermal vascular and metabolic changes similar to those found in chronic venous stasis may play a role in cellulite development.^12^ According to this hypothesis, the precapillary arteriolar sphincters in the affected areas are altered, increasing capillary permeability. Additionally, altered, hyperpolymerized glycosaminoglycans are deposited in the capillary walls, causing pressure on the capillary walls. The combination of increased capillary permeability and pressure on the capillary walls leads to leakage of fluid into the interstitial spaces between the fat lobules and interlobular septa. The ensuing intercellular edema and tissue hypoxia elicit neovascularization and thickening and sclerosis of the fibrous septa, which leads to accentuation of skin irregularities and ultimately the appearance of cellulite.

The inflammation hypothesis for cellulite development arose from the observation of tenderness when pinching the affected skin.^3^ It has been proposed that low-grade septal inflammation may be responsible for dermal atrophy^13^ and that chronic inflammation may play a role in the fibrous septal development.^9^ Inflammation may also be a cause of the endothelial damage seen in cellulite. In proinflammatory states such as obesity and insulin resistance, presence of macrophages, Th1 cells, mast cells, interleukin-6, and tumor necrosis factor alpha can contribute to endothelial damage.^3^

A number of other factors may also contribute to the development or worsen the severity of cellulite (Table 1).^2^ Aging negatively impacts the dermis and the fat lobules. Aging reduces the collagen and elastin content of the dermis, atrophying the dermis. Fat herniation can increase at the subdermal junction through an atrophied dermis. With aging, there is also hypertrophy of fat lobules.^11^ Enlarged fat lobules may cause further imbalances of the biomechanical forces within the subcutaneous layer. Advancing age, thus, increases the risk of cellulite development. Indeed, elderly females with a high BMI have the greatest risk of developing or worsening of cellulite.^10^ Age, however, is unlikely to be a primary contributor because aging of the dermis occurs in both genders.

**Table 1.** Risk Factors for Cellulite^2^

Risk Factors
Gender
Age
Genetics
Race
Increased subcutaneous fat
Diet
Sedentary lifestyle
Pregnancy

Improper diet and lifestyle factors may contribute to the development of cellulite or increase its severity.^14^ High-carbohydrate diets and a sedentary lifestyle can lead to hyperinsulinemia and stimulate lipogenesis. Alcohol consumption also stimulates lipogenesis. Lipogenesis may result in an overall increase in body fat content, thus increasing the risk of developing cellulite. Another important principle is that the fat content difference between high and low BMI is due to the hypertrophy of fat globules (Figures 1, 2). Fat globules or fat cells do not multiply, they undergo hype- or hypotrophy with weight gain or weight loss. During weight gain, the septal network does stretch but does not shrink from weight loss. This is important in patients who have undergone weigh loss as the septal network will be elongated and weak leading to the necessity of more complex procedures addressing both septae and skin.

## PATIENT ASSESSMENT

Clinical evaluation of a patient presenting for cellulite treatment begins with careful assessment of the areas of concern to determine the main contributing or exacerbating factors for cellulite appearance, including skin laxity, dermal atrophy, volume loss, or fat deposition. In addition, there are several conditions that mimic cellulite; thus, the differential diagnosis should not be overlooked. For example, lipoatrophy, generalized edema or lymphedema, and generalized obesity may clinically appear as depressions in the skin.^15^ Using treatments intended for cellulite may lead to an exacerbation of these mimicking conditions.

Clinical evaluation is performed with the patient standing, with legs hip width apart, and not lying down. Standing places a greater tension on the fibrous septa and helps in visualizing the dimples. In the prone position, there is less tension on the fibrous septa and the dimples tend to disappear. Initially, the patient should be standing relaxed, without muscle contraction. This is followed by having the patient contract the underlying muscles and pinching the affected area with the thumb and index finger. Active muscle contraction and pinching accentuate the dimples, making them more visible. The use of handheld lights may also assist in visualizing the dimples.

The severity of cellulite may be graded by using one of several validated scales that are available for this purpose. The Nürnberger–Müller Scale and the Cellulite Severity Scale (CSS) are the most commonly used in clinical trials to evaluate treatment effect. The Nürnberger–Müller Scale uses visual assessment of dimple severity and a pinch test of the skin to categorize skin appearance into 4 grades (0-III; Table 2).^11^ It is easy to administer without the need for a visual tool/scale; but is an unvalidated, purely qualitative scale.^7^

**Table 2.** Nürnberger–Müller Classification^11^

Grade	Cellulite severity description
0	Skin is smooth in both lying down and standing positions
I	Skin is smooth at rest but shows a mattress-like appearance when pinching
II	Skin is smooth at rest but has a mattress-like appearance when standing
III	Skin has a mattress-like appearance in both lying down and standing positions

The CSS, on the other hand, is a validated qualitative and quantitative scale that uses 5 items: number of depressions, depth of depressions, morphology of skin surface alterations, skin laxity, and the Nürnberger–Müller Scale (Table 3).^16^ Each of the items is rated on a scale of 0 to 3. Total scores of 1 to 5 indicate mild cellulite, 6 to 10 indicate moderate cellulite, and 11 to 15 indicate severe cellulite. Although a comprehensive scale, the CSS does not capture the patient perspective.^7^ Also, the multiple ratings may be cumbersome to use in clinical practice and they are not validated for cellulite other than in the buttocks and thighs.

**Table 3.** Cellulite Severity Scale^16^

Item	Cellulite severity description
No. of depressions	0 = no depressions 1 = 1-4 visible depressions 2 = 5-9 visible depressions 3 = 10 + visible depressions
Depth of depressions	0 = no depressions 1 = superficial depressions 2 = medium-depth depressions 3 = deep depressions
Morphology of skin surface alterations	0 = no raised areas 1 = orange peel appearance 2 = cottage cheese appearance 3 = mattress appearance
Skin laxity	0 = absence of laxity 1 = slight draped appearance 2 = moderate draped appearance 3 = severe draped appearance
Nürnberger–Müller classification scale	0 = grade 0 1 = grade I 2 = grade II 3 = grade III

In clinical practice, pretreatment and posttreatment photographs of the affected areas are usually taken to evaluate treatment effects. Patient positioning and lighting are critical components for photographs. Ideally, photographs should be taken without the influence of outside light, since time of day can affect the lighting, and with the same positioning.

## TREATMENT OPTIONS

Many agents/devices targeting various steps/pathways implicated in the etiopathogenesis of cellulite are available to help treat or diminish cellulite appearance, including topical agents, oral treatments, massage, energy-based devices (radiofrequency [RF], laser or light therapy, and acoustic wave therapy), subcision, and injectable treatments (dermal fillers and biologics; Table 4).^2,3,8,9,17,18^ Among these, the earlier treatments, such as topical agents and massage, focused on addressing the underlying impaired microcirculation and drainage deficiencies. But as the understanding of the pathophysiology of cellulite evolved, later treatments, such as subcision, focused on addressing the architectural disturbances in cellulite.

**Table 4.** Types of Treatment for Cellulite

Treatment type
Topical agents
Oral supplements
Massage
Energy-based therapy Radiofrequency Light/laser Acoustic wave
Subcision Manual Vacuum-assisted Laser-assisted Chemical Acoustic
Injectable fillers

### Topical Agents

Topical agents are one of the earliest treatments for cellulite and numerous gels and creams are available for this purpose. They mostly contain a combination of active ingredients, typically methylxanthines (aminophylline, theophylline, and caffeine), retinol, and botanical extracts.^2,3,8,9,17,18^ Stimulation of cutaneous microcirculation, dermal neocollagenesis, and lipolysis; inhibition of lipogenesis, inflammation, and oxidation; and lymphatic drainage and edema reduction are some of the purported effects of the topical agents.

Caffeine and retinol are the most studied ingredients in oral formulations. Caffeine acts by inhibiting phosphodiesterase, thereby inducing lipolysis. It also stimulates cutaneous microcirculation and is an antioxidant.^19,20^ Retinoids act by increasing dermal thickness, increasing angiogenesis, synthesizing new connective tissue components, and increasing the number of active fibroblasts.^17,18^ Placebo-controlled, randomized studies have reported significant improvement in cellulite severity with caffeine and/or retinol-containing topical preparations but these studies were small and of short duration.^21,22^ A systematic review and meta-analysis of topical products for cellulite reduction found a moderate efficacy in thigh circumference reduction.^23^ In the absence of robust data on clinical efficacy and durability of effects, none of the topical formulations currently available and utilized for the treatment of cellulite are approved by the United States Food and Drug Administration (FDA).

### Oral Supplements

A plethora of oral supplements are utilized to improve skin appearance. Supplements containing extracts of *Vitis vinifera*, *Ginkgo biloba*, *Centella asiatica*, *Melilotus officinalis*, *Fucus vesiculosus*, fish oil, and borage oil are thought to be useful in cellulite treatment because of their antioxidant effects.^24^ Aronia juice may help reduce cellulite, as it is believed to enhance cellular metabolism, increase collagen and elastin synthesis, reduce edema and bowel inflammation, and improve microcirculation. There is very little clinical evidence of efficacy with these supplements, and none have received FDA approval. However, significant improvement in cellulite severity and skin appearance was reported in a placebo-controlled study in females with moderate cellulite with oral collagen consumption over a 6-month period.^25^

### Massage

Massage is one of the oldest methods to treat cellulite that works by stimulating lymphatic drainage, thereby addressing the underlying impaired microcirculation and drainage deficiencies associated with cellulite.^26^ Massage can be performed manually or mechanically with the help of devices. Manual massage is rarely performed in clinical practice.

LPG Endermologie (Endo-Systems, LLC, Fort Lauderdale, FL) is an FDA-approved, combined positive and negative pressure, vacuum-assisted mechanical massage system for cellulite treatment.^27,28^ Using positive pressure from 2 rollers and negative pressure from aspiration to the skin and subcutaneous tissue, LPG Endermologie works by causing nonlethal damage to adipocytes which are redistributed to achieve a better skin contour. In observational studies, 15 sessions of 30 to 45 min each twice a week of whole-body endermologie showed significant improvement in cellulite appearance but durability of the effects was not reported. A prospective, randomized trial, however, found it to be only partially better than aminophylline topical cream.^29^

### Energy-Based Therapy

Noninvasive, energy-based devices utilizing RF, light and lasers, and acoustic waves have been extensively studied for the treatment of localized adiposity and/or skin laxity, 2 factors that may contribute to cellulite appearance.

#### Radiofrequency

RF devices deliver thermal energy via electrode(s) to the target area. Thermal energy is produced from the resistance to the flow of an electrical current through the dermis and subcutaneous tissue; this resistance is referred to as bioimpedance. The heat generated elevates the tissue temperature at the target area, stimulating collagen denaturation, remodeling, and neocollagenesis, which cumulatively lead to skin tightening.^8,18^ Depending on the type of device utilized, the therapeutic effect of RF treatment may vary and is dictated by a combination of parameters, including energy density, exposure time, polarity of the radiofrequency, surface cooling, and the method of application.^3^

RF devices are available in various iterations: the first-generation devices include unipolar, monopolar, or bipolar devices and the newer generation devices include multipolar, multigenerator, or temperature-controlled devices.^3,18^ The depth of penetration of the thermic energy differs between the devices, with the multipolar devices having the greatest depth of penetration followed by monopolar and then unipolar devices. Bipolar devices have the least depth of penetration.^30^ In temperature-controlled devices, where superficial skin layers are cooled at the same time, RF can penetrate to the deeper fat tissue and stimulate lipolysis, resulting in circumference reduction.^31^ Some of the RF devices also integrate other technologies, such as infrared light, vacuum suction, and pulsed-electromagnetic fields.^18^

RF devices, particularly the newer generation devices, have been shown to be effective in reducing the appearance of cellulite in clinical studies.^32–35^ Several of these devices have been approved by the FDA for cellulite treatment, including the Velasmooth and Velashape systems (Syneron Medical; Yokne'am Illit, Israel), a bipolar RF device with infrared light and mechanical manipulation of the skin; Exilis Elite (BTL Aesthetics; Newcastle, UK), a monopolar RF device; Venus Legacy (Venus Concept; Ontario, Canada), a multipolar RF device with pulsed magnetic fields; Endymed Body Shaper (Endymed; Freehold, NJ), a multigenerator RF device; ThermiRF (Thermi Aesthetics; Hayward, CA), a novel temperature-controlled RF device with internal probes; Viora Reaction, a bipolar/multipolar RF device with vacuum action.^18^ RF therapy, however, is limited by the need for multiple treatment sessions to see visible results, which are short lived. RF is also associated with bruising although that resolves over time.^3^

#### Light and Laser Therapy

Similar to RF devices, light and laser devices work by emitting thermal energy into the target area.^3,8,17,18^ The extent to which the emitted energy penetrates the target tissue (dermis or subcutaneous tissue) is dependent on the wavelength. The heat generated stimulates collagen remodeling and increases microcirculation, potentially improving cellulite appearance.

The use of light/laser devices, such as intense pulse light; 810 nm diode laser alone or in combination with massage, vacuum, and ultrasound; and long-wave infrared light, for cellulite treatment has not been conclusive.^36,37^ A low-level laser light therapy (LLLT) employing green 532 nm diodes as a stand-alone procedure without massage or mechanical manipulation was found to improve the appearance of cellulite after six 30 min treatments over a 2 week period.^38^ Reduction in at least 1 stage on the Nürnberger–Müller grading scale was seen in about half of the treated patients, which persisted for up to 6 weeks posttreatment. Most LLLT-treated subjects were also satisfied with the improvement in cellulite. The long-pulsed 1064 nm neodymium-doped yttrium-aluminum-garnet (Nd:YAG) laser produced mild-to-moderate improvement in cellulite severity in some subjects after 3 treatments at 4 weeks intervals.^39^ There is, thus, some evidence of benefit for lasers but the need for multiple sessions and the lack of durability of results are limitations.

#### Acoustic Wave Therapy

Acoustic wave therapy is widely used to treat musculoskeletal injuries, as it helps improve cutaneous microcirculation, neocollagenesis, and lymphatic drainage.^40^ Because of these effects, acoustic waves are also used as a treatment for cellulite to help restructure the skin and improve its topography.

Radial shock waves (radial pressure waves) and focused shock waves (extracorporeal shock wave therapy [ESWT]) are the 2 types of acoustic waves used in cellulite treatment. Radial waves diverge as soon as they are discharged and the energy of the waves diminish upon skin penetration and result in low energy diffuse waves at the target site. Because radial waves are low energy, they are generally thought to have superficial tissue effects; but they can penetrate up to 25 mm, thus reaching subcutaneous fat and connective tissue structures.^41^ Focused shockwaves (ESWT), in contrast, are high-energy acoustic waves that penetrate deep into the tissue. The energy of focused shockwaves does not diminish upon skin penetration but converges to achieve maximal energy at the target site.

Cellactor (Storz Medical, Tägerwilen, Switzerland) and Z-wave (Zimmer, Irvine, CA) are the main acoustic wave therapy devices that are used for cellulite treatment. Improvement in cellulite appearance or severity has been reported in several studies^42–46^ but usually 6 to 8 treatment sessions are needed to see a visible reduction.^47^ Data on the durability of results beyond 1 year are lacking.

### Subcision

Subcision is recommended for cellulite depressions present at rest only and not for depressions that are visible with muscle contraction.^9,48^ It is a surgical technique that severs the fibrous septal bands tethering the dermis to the subcutaneous adipose tissue. With the severing of the fibrous septa, biomechanical forces within the subcutaneous layer are redistributed, fat lobules are redistributed into spaces created by the procedure, and fat protrusion is mitigated, resulting in a smoothening of the skin surface. Subcision can be performed manually or it can be vacuum assisted or laser assisted. More recent techniques include chemical subcision, using collagenase enzyme injections, and acoustic subcision.

#### Manual Subcision

Manual subcision is usually performed using a forked cannula or an 18 G noncoring needle inserted to a depth of about 10 to 20 mm into the subcutaneous adipose layer, parallel to the skin surface.^48^ Efficacy was demonstrated in a large retrospective study in subjects with advanced cellulite of the buttocks/thighs.^48^ After a single treatment, almost 80% of subjects were satisfied with their results, which persisted at 2 years follow-up. Notable posttreatment adverse events included excessive elevation of treated areas in 15% of subjects, painful bruising for up to 4 months in 90% of subjects, and hemosiderin pigmentation for up to 10 months in all patients; all of which resolved spontaneously without further treatment.

More recently, a novel manual device (Avéli, Revelle Aesthetics, Inc.; Mountain View, CA) has been developed that utilizes a Targeted, Verifiable Subcision method for selectively identifying and manually releasing the specific septa responsible for causing cellulite depressions in a precise manner.^49^ In an open-label, multicenter study, the device was shown to be effective in improving the appearance of moderate-to-severe cellulite; 95% of subjects achieved a mean ≥1-point reduction in the CSS at 6 months. Adverse events were similar to other manual subcision devices and included mild-to-moderate bruising/ecchymosis in all patients, mild-to-moderate temporary soreness/pain/tenderness in about 50% of patients, and mild edema in about 50% of patients. All adverse events resolved spontaneously without treatment. The device has received FDA approval for the treatment of cellulite on the buttocks and thighs.

Although efficacious, the main drawback of manual subcision is its adverse effects.^48^ In addition, the procedure also has the potential for inconsistent results because it is contingent on the surgeon having the proper skills and technique to perform the subcision. Particularly critical is the surgeon’s ability to maintain the correct depth of 10 to 20 mm. If subcision was to be performed too superficially, excessive elevation or skin necrosis could occur, while too deep a subcision could lead to negligible improvement in the targeted depressions.^9^

#### Vacuum-Assisted Subcision

A vacuum-assisted subcision system has been developed in response to the shortcomings of manual subcision. This FDA-approved system (Cellfina system, Ulthera, Inc.; Mesa, AZ) uses a unique vacuum-assisted tissue capture platform to provide precise control of the depth and area of release for repeatable durable results.^15,50^ Treatment depth (6 or 10 mm) and area (5 cm or 3 × 6 cm) are adjustable and user selectable. Tissue release is accomplished through the mechanical action of a reciprocated razor-thin (0.45 mm) microblade. The system also enables controlled infiltration of tumescent anesthetic solution at the precise depth of the intended release with an integrated 3 inch 22 G multiple side-hole needle.

The safety and efficacy of this vacuum-assisted system were demonstrated in a multicenter clinical study.^15,50^ After 1 treatment, at 1-year follow-up, 93% of subjects with moderate to severe cellulite had ≥1 point improvement in the CSS and 94% were satisfied with their results. Most common adverse effects were ecchymosis/bruising, edema, areas of palpable softness, and soreness. All adverse events were mild in severity, of short duration, and resolved spontaneously. Results were durable, lasting >3 years.^15^

#### Laser-Assisted Subcision

In laser-assisted subcision, targeted disruption of subcutaneous fibrous septa is performed with percutaneous subdermal delivery of laser energy. The best evidence on the safety and effectiveness of this modality comes from the FDA approved, minimally invasive, 1440 nm Nd:YAG with a 1000 m side-firing fiber laser at 8 to 10 W (Cellulaze system; Cynosure, Inc.; Westford, MA). In a multicentered study, a single treatment of the thighs and/or buttocks with the 1440 nm Nd:YAG laser showed sustainable improvement in the appearance of cellulite for at least 1 year in 90% of the treatment sites.^51^ High treatment satisfaction was also reported by at least 90% of physicians and subjects at 6 months.^52^

#### Chemical Subcision

Collagenase isolated and purified from *Clostridium histolyticum* selectively hydrolyzes the triple helical region of collagen and is FDA approved for the treatment of collagen-associated disorders (such as Peyronie’s disease and Dupytren’s contracture).^53^ Collagenase Clostridium histolyticum (CCH) comprises 2 collagenases, Collagenase I (AUX-I, Clostridial class I collagenase) and Collagenase II (AUX-II; Clostridial class II collagenase), in a 1:1 ratio. The 2 collagenases have different specificities but work synergistically providing broad collagen hydrolyzing reactivity. CCH can act on subdermal collagen found in the fibrous septa involved in the development of cellulite.

In phases 2 and 3 randomized, placebo-controlled trials, CCH significantly improved cellulite appearance in females with moderate-to-severe cellulite of the buttocks or posterolateral thighs.^54,55^ A total of 0.84 mg of CCH was administered subcutaneously in three 0.1 mL aliquots in 3 treatment sessions approximately 21 days apart. Improvement was durable through 2 years of follow-up.^56^ CCH was generally well tolerated. The most common adverse events were injection-site bruising and pain.

In 2020, the FDA approved a CCH preparation (Qwo, Endo Aesthetics LLC; Malvern, PA) for the treatment of moderate-to-severe cellulite of the buttocks in adult females.^57^ However, on December 6, 2022, the manufacturer announced the termination of the production and sale of Qwo due to concerns about the extent and variability of bruising following initial treatment as well as the potential for prolonged skin discoloration.^58^

#### Acoustic Subcision

In acoustic subcision, rapid acoustic pulses are used to disrupt fibrous septae and stimulate neocollagenesis through acoustic shearing.^59^ Rapid acoustic pulses are generated using a new technology (Rapid Acoustic Pulse technology, Allergan Aesthetics; Irvine, CA). The acoustic wave device using this technology (RESONIC, Allergan Aesthetics), has been approved by the FDA for long-term improvement in cellulite appearance up to 1 year.^60^ The device, when placed on the skin and activated, emits high-frequency acoustic waves up to 50 times per second, which reportedly physically change the fibrous septa while reestablishing a smooth skin topography. Results are seen after 1 treatment session lasting less than an hour. In an open-label, multicenter study, all subjects had a mean CSS reduction of 1.01 (a 29.5% reduction from baseline) at 12 weeks after a single treatment.^59^ Skin laxity was graded as improved, much improved, or very much improved in 67% of treated areas and satisfaction rate was 93% among study subjects. No unexpected or serious adverse events were noted at treatment or follow-up. Overall average pain score during treatment was 2.4 (0–10 pain scale) and 0.3 immediately posttreatment. Clinical improvements in cellulite were seen for up to 12 months and subjects are continued to be followed up to ascertain long-term durability of outcomes.^61^

### Injectable Treatments

There are 2 types of injectable treatments: injectable biologics and injectable fillers. The injectable biologic, collagenase from *C. histolyticum*, was reviewed under the Chemical Subcision section. The injectable fillers, calcium hydroxylapatite and poly-l-lactic acid, are reviewed in this section.

#### Injectable Fillers

Calcium hydroxylapatite (CaHA; Radiesse; Merz North America, Inc.; Raleigh, NC) is currently approved for the correction of moderate-to-severe facial wrinkles and folds, the correction of volume loss in the dorsum of the hands, and the improvement of moderate-to-severe loss of jawline contour.^62^ In its undiluted form, it is highly viscous and is used as a volumizing agent.^63^ When diluted 1:2 or more, it loses its viscoelastic properties and ceases to be a volumizing agent. In this diluted state, CaHA is a suspension of microspheres, which when injected subcutaneously can be distributed over a large surface area. Diluted CaHA induces neocollagenesis and elastogenesis which can lead to increased dermal thickness, skin elasticity, and pliability.^64^ Based on these beneficial skin effects, diluted CaHA has been used as a treatment for cellulite. In a retrospective study, a single treatment of diluted CaHA in combination with microfocused ultrasound with visualization was found to significantly improve cellulite severity and skin laxity from baseline in females with moderate-to-severe cellulite with high subject satisfaction. Both treatments were well tolerated.^65^

Poly-l-lactic acid (PLLA; Sculptra, Galderma Laboratories, L.P.; Fort Worth, TX) is an injectable volumizer. It is FDA approved for the correction of shallow to deep nasolabial fold contour deficiencies and other facial wrinkles for use in immunocompetent subjects and for the restoration/correction of HIV-associated facial lipoatrophy.^66^ PLLA is also a biostimulator that stimulates neocollagenesis, leading to skin tightening. PLLA treatments (3 treatments every 4 weeks), in combination with subcision, have been shown to significantly improve cellulite severity.^67^ In another study, subcision followed by PPLA treatments in the same treatment session improved cellulite-associated flaccidity, which lasted over a 2-year follow-up period.^68^ Subject satisfaction was high in both studies.

## TREATMENT ALGORITHM

**Figure 4.**
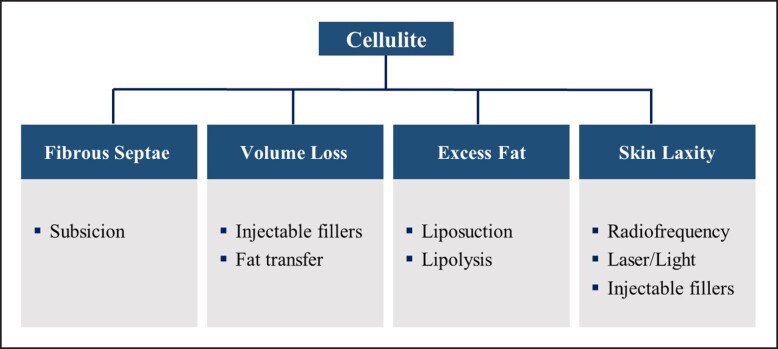
Cellulite treatment algorithm. Treatment is based on the underlying structural alteration of the fibrous septa and/or the exacerbating factors that worsen cellulite appearance–volume loss, excess fat, and skin laxity.

Cellulite treatment should be individualized and address the underlying structural alteration of the fibrous septa and/or the exacerbating factors that worsen cellulite appearance–volume loss, excess fat, and skin laxity (Figure 4). The goal is to address the anatomic disturbances in each layer: the dermis, adipose tissue, and connective tissue. Often, >1 treatment modality may be needed to address the underlying contributing factors. Combining treatments that target different tissue planes can be safely performed on the same day. However, consideration should be given to scheduling treatments on different days per patient comfort or if there is a perceived increased risk to the patient.

**Figure 5.**
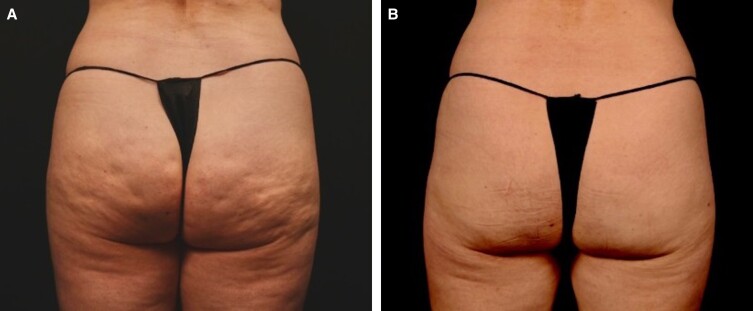
A 46-year-old female patient (A) prior to and (B) 9 months after cellulite treatment by manual subcision using the new FDA-approved device (Avéli; Revelle Aesthetics, Inc.). Note, no prior procedures had been performed on the buttocks.

**Figure 6.**
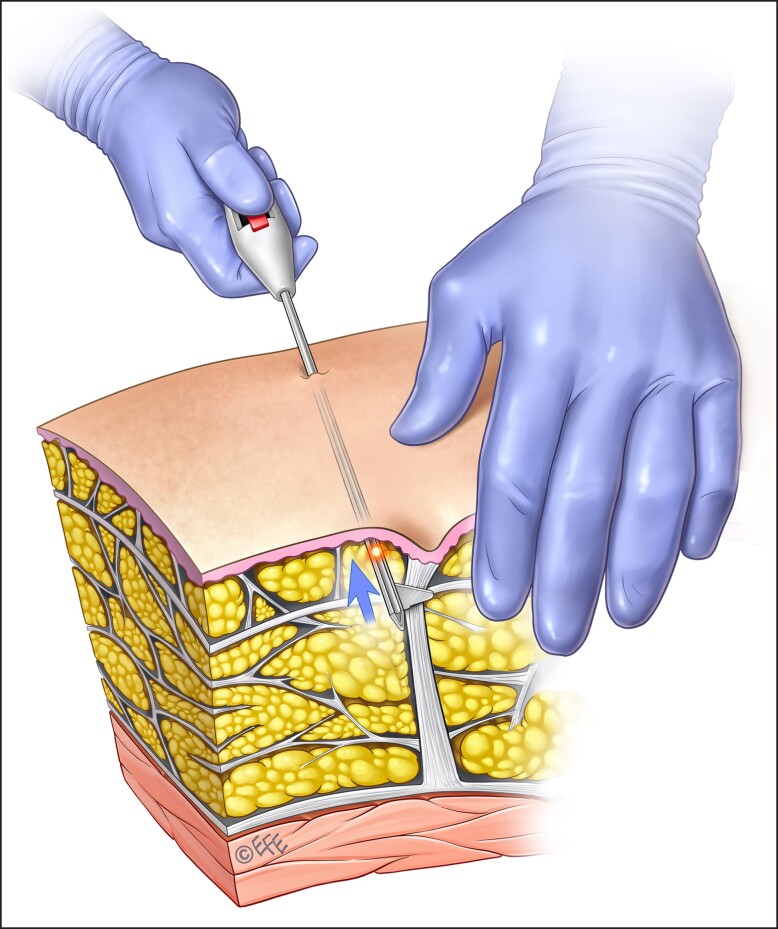
Illustration of the new FDA-approved device (Avéli; Revelle Aesthetics, Inc.) used to treat the patient shown in Figure 5. Artwork created by and published with permission from Dr Levent Efe, CMI.

Subcision is reserved for the treatment of severe cellulite, that is, for cellulite depressions/dimples present at rest only. Prior to this procedure, tumescent fluid is percutaneously infiltrated within the target area to elevate the subcutaneous tissue from the underlying vital structures and create a plane for the safe performance of subcision and anesthetize the area.^9^ The author's preferred technique is manual subcision using the new FDA-approved device (Figures 5, 6). The septae, as previously described, vary in thickness and orientation and thus difficult to assess clinically. Therefore, the device utilized should have the immediate ability to confirm if the “dimpling” is released. Utilizing a device that releases and confirms the release of the septae is important for more predictable outcomes. It is also important to keep in mind that additional procedures to address skin quality or volume change may have to be addressed in subsequent procedures. Patients should be made aware of the potential need for multiple treatment modalities to address the multiple etiologies of cellulite.

In addition to treatment, all patients should be advised to undergo lifestyle modifications and/or maintain a healthy lifestyle that should include a low fat, low sodium diet; regular exercise; and smoking and alcohol restriction.^3,14^ Excessive weight gain from an improper diet and a sedentary lifestyle can exacerbate cellulite appearance.^14^ At the same time, excessive weight loss should also be avoided as this can lead to skin laxity and worsen cellulite appearance.

## CONCLUSIONS

Cellulite is an aesthetically distressing skin condition mostly affecting females. Multiple therapeutic approaches are available but many only ameliorate cellulite appearance or reduce its severity. Treatments that target the fibrous septa appear to produce more significant improvement that is more durable. Whether these latter treatments can completely “cure” cellulite or prevent its recurrence remains to be seen. Further elucidation of the etiopathogenesis and pathophysiology of cellulite is needed to produce more targeted therapies in the future. For the present, a treatment strategy that utilizes a combination of modalities targeting the multiple etiologies of cellulite might produce the best results.
